# The relationships within the Chaitophorinae and Drepanosiphinae (Hemiptera, Aphididae) inferred from molecular-based phylogeny and comprehensive morphological data

**DOI:** 10.1371/journal.pone.0173608

**Published:** 2017-03-13

**Authors:** Karina Wieczorek, Dorota Lachowska-Cierlik, Łukasz Kajtoch, Mariusz Kanturski

**Affiliations:** 1 Department of Zoology, Faculty of Biology and Environmental Protection, University of Silesia, Katowice, Poland; 2 Department of Entomology, Institute of Zoology, Jagiellonian University, Kraków, Poland; 3 Institute of Systematics and Evolution of Animals, Polish Academy of Sciences, Kraków, Poland; Natural Resources Canada, CANADA

## Abstract

The Chaitophorinae is a bionomically diverse Holarctic subfamily of Aphididae. The current classification includes two tribes: the Chaitophorini associated with deciduous trees and shrubs, and Siphini that feed on monocotyledonous plants. We present the first phylogenetic hypothesis for the subfamily, based on molecular and morphological datasets. Molecular analyses were based on the mitochondrial gene cytochrome oxidase subunit I (*COI*) and the nuclear gene elongation factor-1α (*EF-1α*). Phylogenetic inferences were obtained individually on each of genes and joined alignments using Bayesian inference (BI) and Maximum likelihood (ML). In phylogenetic trees reconstructed on the basis of nuclear and mitochondrial genes as well as a morphological dataset, the monophyly of Siphini and the genus *Chaitophorus* was supported. *Periphyllus* forms independent lineages from *Chaitophorus* and Siphini. Within this genus two clades comprising European and Asiatic species, respectively, were indicated. Concerning relationships within the subfamily, *EF-1α* and joined *COI* and *EF-1α* genes analysis strongly supports the hypothesis that Chaitophorini do not form a monophyletic clade. *Periphyllus* is a sister group to a clade containing *Chaitophorus* and Siphini. The Asiatic unit of *Periphyllus* also includes *Trichaitophorus koyaensis*. The analysis of morphological dataset under equally weighted parsimony also supports the view that Chaitophorini is an artificial taxon, as *Lambersaphis pruinosae* and *Pseudopterocomma hughi*, both traditionally included in the Chaitophorini, formed independent lineages. *COI* analyses support consistent groups within the subfamily, but relationships between groups are poorly resolved. These analyses were extended to include the species of closely related and phylogenetically unstudied subfamily Drepanosiphinae, which produced congruent results. Genera *Drepanosiphum* and *Depanaphis* are monophyletic and sister. The position of *Yamatocallis tokyoensis* differs in the molecular and morphological analyses, i.e. it is either an independent lineage (*EF-1α*, *COI*, joined *COI* and *EF-1α* genes) or is nested inside this unit (morphology). Our data also support separation of Chaitophorinae from Drepanosiphinae.

## Introduction

The aphid subfamily Chaitophorinae (Hemiptera: Aphididae) comprises about 170 species within 12 genera traditionally divided into two tribes–Chaitophorini and Siphini [1–2]. These tribes overlap with two bionomic groups. The tribe Chaitophorini live on deciduous trees and shrubs. The genera *Chaitophorus* (about 90 species), *Chaitogenophorus* (one species), *Lambersaphis* (one species) and *Pseudopterocomma* (two species) are associated with Salicaceae–*Populus* spp. (poplar or aspen) or *Salix* spp. (willow). The genera *Periphyllus* (about 42–44 species), *Trichaitophorus* (six species) and *Yamatochaitophorus* (two species) are associated with Sapindaceae, mostly with *Acer* spp. (maple); a few species of *Periphyllus* feed on *Aesculus* or *Koelreuteria* [3–6]. The aphids usually form colonies on young leaves, leaf stems or petioles, young shoots or branches of their host plants. Exceptionally, some of the species of *Chaitophorus* and *Pseudopterocomma* feed on the roots and subterranean parts of trunks or one-year-old branches of the host plants [4]. The tribe Siphini, on the other hand (genera *Atheroides*, *Caricosipha*, *Chaetosiphella*, *Laingia* and *Sipha*, with two subgenera *Sipha* s.str. and *Rungsia*), feed on Poaceae, Cyperaceae, Juncaceae or Typhaceae [7–8]. Most of the species of Siphini live on the leaves, rarely on steams or inflorescences, forming dense colonies or feeding singly. Some species (e.g. *Laingia psammae* Theobald, 1922, or *S*. *(Rungsia) maydis* Passerini, 1860) can live at ground level, but never feed on the underground parts of their host plants [8]. Biology and the life cycle of some of the species in this subfamily are unknown, however colonies of most species of Chaitophorinae are usually ant-attended. Almost all species are known to be holocyclic, with a sexual phase in autumn. However, in some regions where winters are mild, *S*. *(Sipha) flava* (Forbes, 1884) and *S*. *(R*.*) maydis* do not produce sexual forms and are anholocyclic, reproducing parthenogenetically throughout the year [8–9]. This group of aphids is also monoecious, i.e. they do not host alternate, and very host specific. Chaitophorinae are so far mainly recorded from the Holarctic, with about 140 species distributed in the Palaearctic region and 30 native to the Nearctic. Siphini and the genus *Periphyllus* are predominantly Palaearctic, with just six species native to North America. *Lambersaphis*, *Chaitogenophorus*, *Trichaitophorus* and *Yamatochaitophorus* occur only in Central or East Asia. The most numerous genus, *Chaitophorus*, is widely distributed both in the Palaearctic and Nearctic, whereas *Pseudopterocomma* is native to North America [10–12].

The literature indicates that for a long time Chaitophorinae has not been regarded as a homogenous group. In 1915, van der Goot [13], in his classification of aphids, for the first time placed the genera *Chaitophorus*, *Chaitophorinella* (= *Periphyllus*) and *Sipha* in the Chaithophorina. In 1944 Börner [14] divided the family Chaitophoridae (Börner’s Chaetophoridae) into two subfamilies—Chaitophorinae (with the tribes Chaitophorini and Periphyllini) and Siphinae (with genera *Atheroides*, *Caricosipha*, *Laingia* and *Sipha*). The same system of classification of this group of aphids was followed by Börner [15] and Börner and Heinze [16]. In 1948, Mordvilko [17], unlike in Börner’s classification, placed the genera *Atheroides*, *Laingia* and *Sipha* (subtribe Siphea) in the subfamily Phyllaphidinae and tribe Phyllaphidini, but left the genera *Chaitophorus* and *Periphyllus* in the subfamily Chaitophorinae. Shaposhnikov [18] improved Mordvilko’s system by distinguishing two subfamilies within the Chaitophoridae: Chaitophorinae (with two tribes: Chaitophorini and Periphyllini) and Atheroidinae (= Siphinae). In the 1960s two new genera were erected—*Lambersaphis* by Narzikulov [19] and *Pseudopterocomma* by MacGillivray [20], both closely related to Chaitophorini. The similarity of the genus *Trichaitophorus* to *Chaitophorus* and *Periphyllus* was pointed out by Hille Ris Lambers and Basu [21]. Higuchi [22] erected *Yamatochaitphorus*, and also included this genus in the Chaitophorini, with a close relationship with *Trichaitophorus*. All these genera, grouped into the two tribes Chaitophorini and Siphini, were listed in the classification of aphids by Remaudière and Remaudière [1] and this division is now generally accepted. The last genus to be incorporated into the Chaitophorini was *Chaitogenophorus* [2].

The question is whether this classification reflects the evolution of this group of aphids. A high level of polymorphism, morphometric differences in the spring and autumnal viviparous generations of the same species, connected with the presence in the life cycle of aestivating morphs (dimorphs) (e.g. *Periphyllus*, *Trichaitophorus*) or cryptic mode of life (e.g. Siphini) make the Chaitophorinae a difficult subject for study and the main reason for the taxonomic confusion in this subfamily [8,23,24]. Altogether Chaitophorinae have been previously studied morphologically and local faunas reviewed [25,10,26,11,27], only the Palaearctic species in the genus *Chaitophorus* have been revised [28] and a monograph on the tribe Siphini published [8]. Data on the relationships within Chaitophorinae are rare [29–31], including cladistics analyses [8]. No phylogenetic studies on the Chaitophorinae as a whole have been published. The phylogenetic trees based on nuclear and mitochondrial genes [32–35] or the DNA of the obligate symbiont *Buchnera aphidicola* [36] included limited sampling of Chaitophorinae (five of the 170 described species). As these studies focused on higher relationships within Aphididae and only one species of the genera *Periphyllus* and *Chaitophorus* or *Chaitophorus* and *Sipha* (never combined representatives of both Chaitophorini and Siphini) were studied, the tribal status is untested. Wieczorek and Kajtoch [37] on the other hand, explored the phylogeny of Siphini using molecular data together with morphological and biological characters, and included the genera *Periphyllus* and *Chaitophorus* as outgrups. In this paper the monophyly of Siphini was confirmed, however the Chaitophorini did not form a monophyletic lineage. The unexpected result of these studies is that *Chaitophorus* may be more closely related to the monocotyledonous feeding Siphini than the Acer feeding genus *Periphyllus*. Thus, it is now important to test the monophyly and major relationships of Chaitophorinae using a broad taxonomic sample and analyzing mitochondrial and nuclear genes, morphological and biological dataset.

In the present paper, we also extend our analysis by including the Drepanosiphinae (with one tribe the Drepanosiphini), which is another poorly studied subfamily of aphids, closely related to Chaitophorinae. Drepanosiphinae comprises five genera and about 40 species, all associated with *Acer* spp. The genera *Drepanaphis* (17 species) and *Shenahweum* (one species) are Nearctic, genera *Drepanosiphoniella* (four species) and *Drepanosiphum* (nine species) are west Palaearctic, whereas the genus *Yamatocallis* (eight species) is distributed in eastern Asia. The aphids live on leaves, usually not in dense colonies and are not attended by ants. All known species are monoecious and holocyclic [4–6]. Among Drepanosiphinae the interspecific relationships within *Drepanaphis* are known [38]. Recently, the taxonomic revisions of *Shenahweum* [39], *Drepanosiphoniella* [40] and *Drepanosiphum* [41] have been published. Although *Drepanosiphum platanoidis* (Schrank, 1801) is a model species in numerous studies on the ecology of aphids [42–47], relationships within this subfamily are unstudied. The collective evidence from aphid parasites [48], morphology of extant taxa [49–50] and fossils [51] indicate that Drepanosiphinae and Chaitophorinae are sister groups. Molecular data, however poorly sampled as only two species of Drepanosiphinae were studied, supports this view [32–34], or even indicate that the Drepanosiphinae and Chaitophorinae could be combined in a single unit [52].

Chaitophorinae and Drepanosiphinae are one of the major lineages within the Aphididae. It is important to determine the relationships within as well as between particular genera in these subfamilies. This analysis presents the first phylogenetic hypothesis including both subfamilies. Compared with our previous study [37], an expanded dataset was used to test: (1) whether Siphini and Chaitophorini are mutually monophyletic within the subfamily Chaitophorinae and Drepanosiphini within Drepanosiphinae; (2) the taxonomic positions of some genera and their redefinition; (3) the hypothesis that Drepanosiphinae and Chaitophorinae could be combined into a single unit. We used sequences obtained from the mitochondrial gene cytochrome oxidase subunit I (*COI*) and the nuclear gene elongation factor-1α (*EF-1α*), supplemented by 91 morphological and biological characters, to reconstruct the relationships between these aphids.

## Materials and methods

### Molecular data

#### Taxon sampling

**Sequenced taxa**. We obtained molecular data for a total of 36 species. The specimens used for molecular studies were preserved in 99.8% ethanol. Specimens from the same clones were also preserved in 70% ethanol for producing slides of voucher specimens and identification. The specimens were mounted in Berlese liquid on slides. Voucher specimens for each sample were identified by K. Wieczorek based on morphological diagnostic features using standard literature-based keys and a comparison with previously identified specimens kept in the University of Silesia, Department of Zoology, Katowice, Poland (UŚ). All samples and voucher slides were also deposited in the collection of UŚ. Most sequences were directly obtained from the collected specimens. Details of the sequenced taxa, voucher information and numbers of GenBank sequences for all the species studied (both downloaded and newly submitted) are presented in S1 Table.

**Ingroup**. 25 species belonging to eight genera of Chaitophorinae were included in this study. All genera of Siphini (five) and three (of seven total genera) of the larger genera of Chaitophorini were sampled. There are only one or two rare species in each of the genera *Lambersaphis*, *Yamatochaitophorus*, *Chaitogenophorus* and *Pseudopterocomma* and they occur only at a few locations and therefore they were not included in the molecular study.

**Outgroup**. 11 species of six subfamilies (Aphidinae, Calaphidinae, Drepanosiphinae, Hormaphidinae, Lachninae, Phyllpahidinae) were chosen to serve as outgroups. Among them six species of Drepanosiphinae belonging to three of the five genera were also selected, because Drepanosiphinae is the sister group of Chaitophorinae in view of historical taxonomy of Aphididae. Molecular analyses based on the mitochondrial gene cytochrome oxidase subunit I (*COI*) were rooted with *Hamamelistes betulinus* Horvath, 1896 (Hormaphidinae) and *Eulachnus brevipilosus* Bӧrner, 1940 (Lachninae), whereas molecular analyses based on the nuclear gene elongation factor-1α (*EF-1α*) were rooted with *Aphis (Aphis) craccivora* Koch, 1854, *Uroleucon (Uromelan) jaceae* (Linnaeus, 1758) (Aphidinae) and *Clethrobius comes* (Walker, 1848) (Calaphidinae).

**Genes**. Molecular analyses were based on the mitochondrial gene cytochrome oxidase subunit I (*COI*) and the nuclear gene elongation factor-1α (*EF-1α*). Based on previous molecular studies on aphids, a mitochondrial gene was selected to provide resolution at lower taxonomic levels (generic and specific) [53–56], whereas a nuclear gene was used to provide a deeper resolution within the subfamily [57–60].

**DNA extraction, amplification and sequencing**. Genomic DNA was extracted from one to three individuals from the same colony of each species using the NucleoSpin Tissue Kit (Macherey-Nagel, Germany). Amplification of the partial mitochondrial *COI* and the nuclear *EF-1α* was done using the following pairs of primers, respectively: LepF and LepR [61] and Shirley and Prowler [62]. PCR was done in 30-μL reaction volumes with 3.0 μL of 10 × PCR buffer, 3.0 μL of 25 mm MgCl2, 0.6 μL of deoxynucleotide triphosphate (dNTP) mixture, each in a 10 mm concentration, 0.6 μL of each 15 mm forward and reverse primers, 1.0–3.0 μL of 100 ng of genomic DNA, 0.2 μL of Taq DNA polymerase and sterile and de-ionized water (up to 30.0 μL). The cycling profile for the PCR was as follows: 95°C for 4 min, 35 cycles of 95°C for 30 s, 50°C (for *COI*) 55°C (for *EF-1α*) for 1 min, 72°C for 2 min and a final extension period at 72°C for 10 min. After purification (NucleoSpin Extract II (Macherey-Nagel)), the PCR fragments were sequenced using a BigDye Terminator v.3.1. Cycle Sequencing Kit (Applied Biosystems, Foster City, CA, USA) and ran on an ABI 3100 Automated Capillary DNA Sequencer.

**Sequence edition and alignment**. Sequences were checked and aligned using BIOEDIT v.7.0.5.2 [63] and CLUSTALX [64]. No stop codons or indels that would indicate the presence of nuclear pseudogenes were found in the mitochondrial protein-coding genes. Introns in *EF*-*1α* sequences contained a large number of variably sized indels, which were removed prior to further analysis. All sequences were deposited in GenBank, and accession numbers are given in S1 Table. The Akaike Information Criterion in MrModeltest 2.3 [65] in conjunction with PAUP* [66] was used to determine the best-fitting nucleotide substitution model. The GTR+I+G model was chosen for *COI* (proportion of invariable sites I = 0.5890, gamma distribution shape parameter G = 1.1591), the GTR+I+G model for *EF*-*1α* (proportion of invariable sites I = 0.5035, gamma distribution shape parameter G = 0.7386) and GTR+I+G for joined *COI* and for *EF*-*1α* alignments (proportion of invariable sites I = 0.5049, gamma distribution shape parameter G = 0.7605). The final dataset used for phylogenetic analyses contained 634 bp of *COI* and 458 bp of *EF*-*1α*. As the sequences generated from individuals from a single colony were identical (for all species we recorded only single haplotypes for *COI* and *EF*-*1α*), for further analyses only a single sequence of each gene was used. We managed to obtain *COI* sequences for 27 species and *EF*-*1α* sequences for 31 taxa. The coverage of species using both markers was only partial due to difficulties in obtaining PCR products for some species, mainly when using *COI* primers. Attempts to use standard barcode primers (LCO1490 and HCO2198; [67]) also failed to generate amplicons for these species.

### Morphological and biological data

#### Taxon sampling

**Fig 1:**
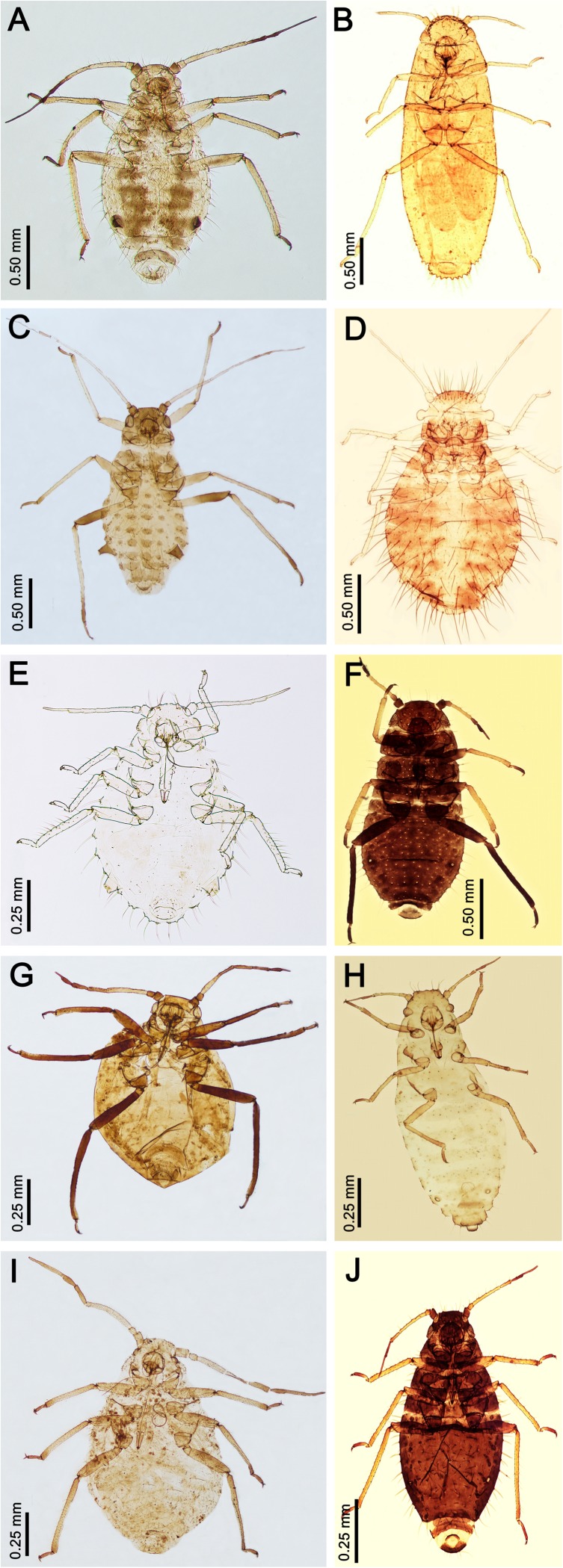
Chaitophorinae—dorsal view (apterous viviparous females). (A) *Chaitophorus leucomelas*; (B) *Atheroides serrulatus*; (C) *Periphyllus testudinaceus*; (D) *Caricosipha paniculatae*; (E) *Trichaitophorus koyaensis*; (F) *Chaetosiphella stipae*; (G) *Lambersaphis pruinosae*; (H) *Laingia psammae*; (I) *Pseudopterocomma hughi*; (J) *Sipha* (*Rungsia*) *maydis* LM.

**Ingroup**. 29 species belonging to all genera of Chaitophorinae (with exception of *Chaitogenophorus* for which there were no samples) were included in this study (Fig 1A–1J).

**Fig 2:**
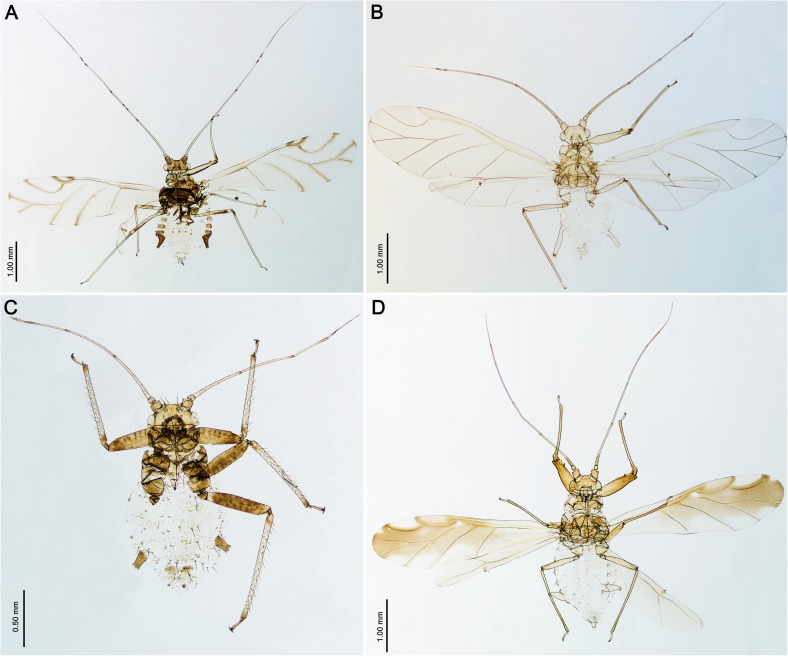
**Drepanosiphinae—dorsal view (A, B, D alate viviparous females; C apterous female).** (A) *Drepanosiphum platanoidis*; (B) *Drepanaphis acerifoliae*; (C) *Drepanosiphoniella aceris*; (D) *Yamatocallis tokyoensis* LM.

**Outgroup**. 14 species of six subfamilies (Aphidinae, Calaphidinae, Drepanosiphinae, Hormaphidinae, Lachninae, Phyllahpidinae) were chosen as outgroups. Among them eight species covering all the genera of Drepanosiphinae, were also used in this study (Fig 2A–2D).

**Morphological and biological characters**. A total of 91 characters were scored for 43 species, including 83 morphological and eight biological characters. Morphological characters were evaluated for viviparous (apterous and alate females) and sexual (oviparous females and males) generations of the species studied (from five to 15 individuals). The characters used for the morphological analysis include previously published characters [8,37] as well as a number of newly developed ones. Characters of specimens viewed under a Nikon Ni-U light microscope and photographed using a Nikon DS-Fi2 camera or a scanning electron microscope were scored. For the SEM photographs, individuals collected in the field were preserved in 70% ethanol for several days and prepared following a mofidied Kanturski et al. [68] method as follows. The samples were transferred into 6% phosphotungstic acid (PTA) solution in 70% ethanol for 24 hours. Dehydration was achieved by using a graded ethanol/water series of 80%, 90% and 96% with 20 minutes at each concentration and 30 minutes in two changes of absolute ethanol. Dehydrated specimens were subsequently dried in 1:3, 1:2 and 2:3 ratio solutions of hexamethyldisilazane (HMDS) in absolute alcohol for 30 minutes and two changes in undiluted HMDS. Samples were mounted on aluminum stubs using double-sided adhesive carbon tape and sputter-coated in a Pelco SC-6 sputter coater (Ted Pella Inc., Redding, CA, USA) to obtain a layer thickness of about 25 nanometers. The samples were imaged using a Hitachi SU8010 field emission scanning electron microscope FESEM (Hitachi High-Technologies Corporation, Tokyo, Japan) at 5.0 and 7.0 kV accelerating voltage with a secondary electron detector (ESD).

Specimens were borrowed from the following scientific collections (preceded by acronyms used in this paper): BMNH–the Natural History Museum, London, UK; MNHN–Muséum national d’Histoire naturelle, Paris, France; UŚ –Department of Zoology, University of Silesia, Katowice, Poland; ZMPA–Zoological Institute of the Polish Academy of Sciences, Warsaw, Poland. Details of the all species studied are presented in S1 Table. The complete matrix is presented in S2 Table and S3 Table.

Descriptions of character are as follows:

#### Apterous viviaparous females

**Fig 3:**
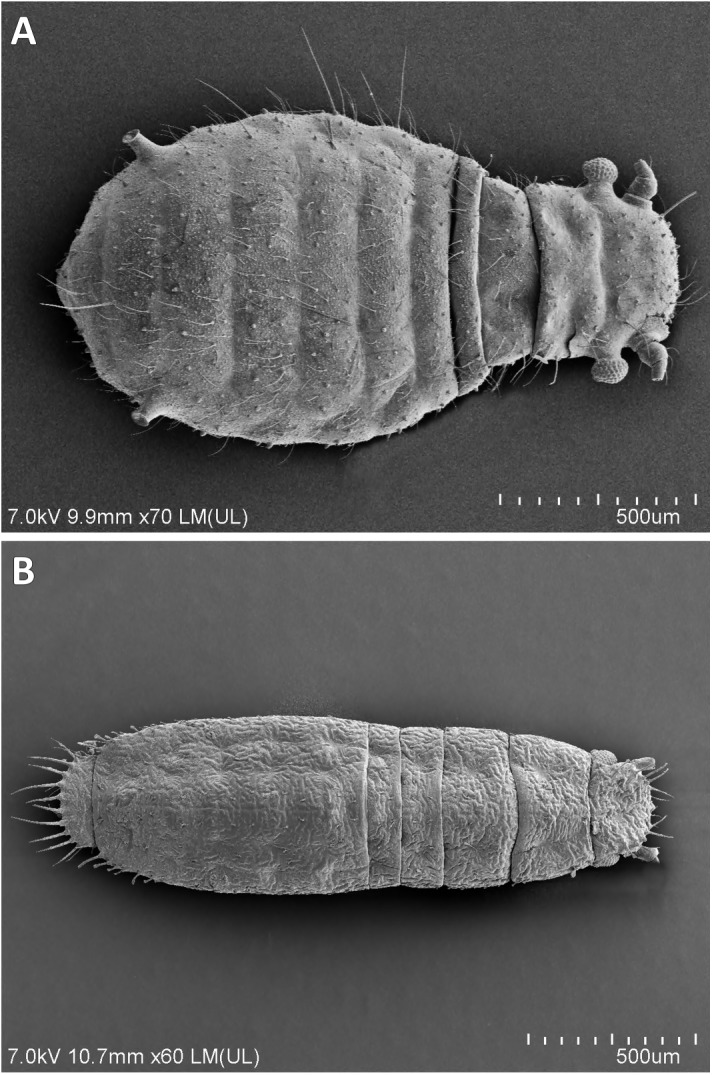
Shape of body (apterous viviparous females). (A) pear-shaped *Caricosipha paniculatae*; (B) slender *Atheroides serrulatus* SEM.

0 Type of body: (0) oval or pear-shaped (Fig 3A); (1) slender (Fig 3B)

**Fig 4:**
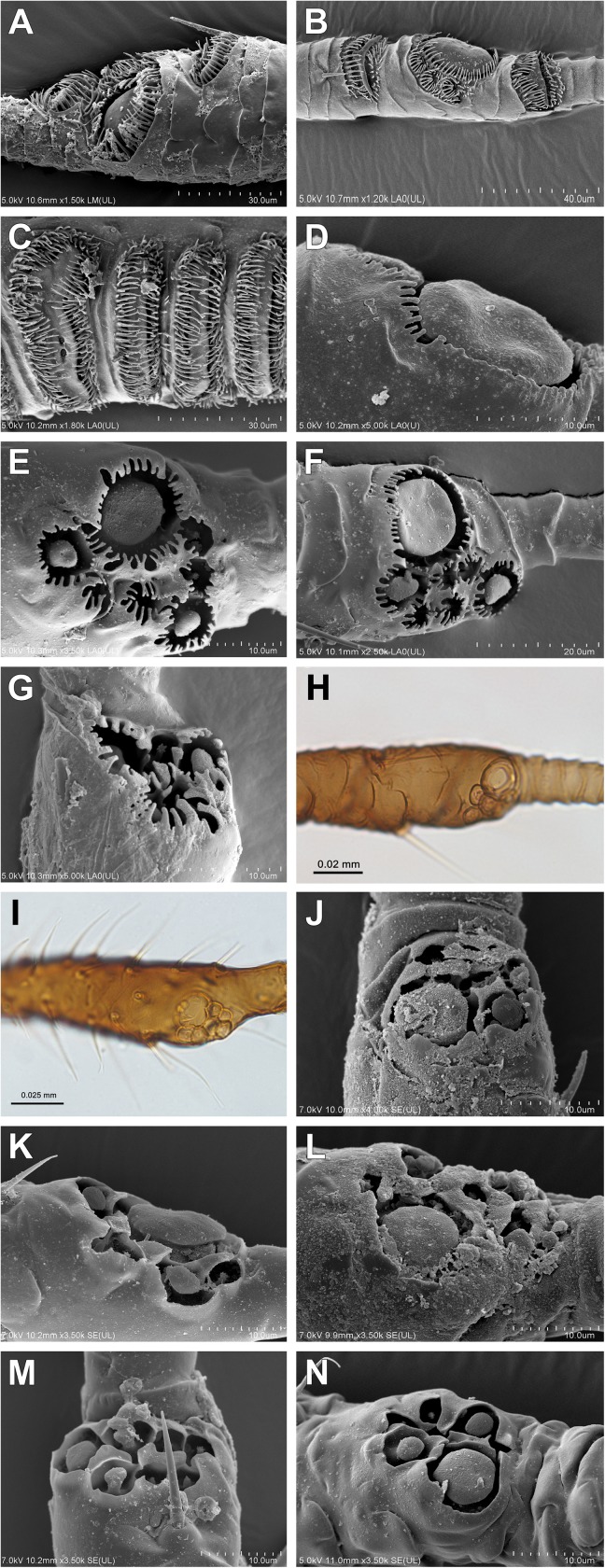
Rhinaria. (A-C) Ciliated (alate viviparous females): (A) *Drepanosiphum platanoidis*; (B) *Drepanaphis acerifoliae*; (C) *Yamatocallis tokyoensis* SEM. Not ciliated (apterous viviparous females): (D) *Drepanosiphoniella aceris* Drepanosiphinae; (E) *Chaitophorus populeti*; (F) *Periphyllus testudinaceus*; (G) *Trichaitophorus koyaensis* SEM; (H) *Lambersaphis pruinosae*; (I) *Pseudopterocomma hughi* LM; (J) *Atheroides serrulatus*; (K) *Caricosipha paniculatae*; (L) *Chaetosiphella stipae*; (M) *Laingia psammae*; (N) *Sipha* (*Rungsia*) *maydis* Chaitophorinae SEM.

1 Aleyrodiform: (0) absent; (1) present
  2 Frons: (0) without tubercles; (1) with lateral or frontal tubercles
3 Number of antennal segments: (0) 6; (1) 5 or 4; (2) 3
4 Primary rhinaria: (0) ciliated; (1) not ciliated (Fig 4D–4N)

**Fig 5:**
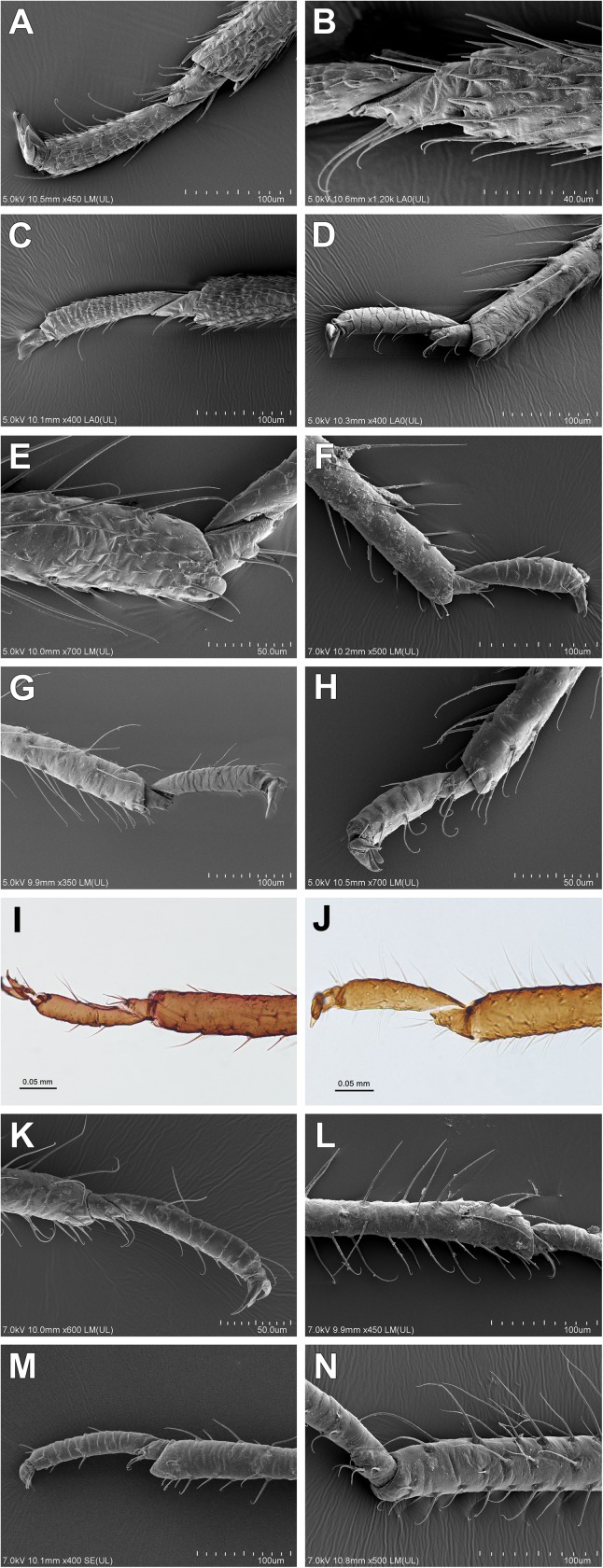
Characters of tibiae. (A-C) Spinules and rastral spines present on distal part of tibiae (alate viviparous females): (A) *Drepanosiphum platanoidis*; (B) *Drepanaphis acerifoliae*; (C) *Yamatocallis tokyoensis* Drepanosiphinae SEM. (D-F) Only spinules present on distal part of tibiae (apterous viviparous females): (D) *Drepanosiphoniella aceris* Drepanosiphinae; (E) *Periphyllus testudinaceus*; (F) *Caricosipha paniculatae* Chaitophorinae SEM. (G-N) Distal part of tibiae smooth (apterous viviparous females): (G) *Chaitophorus populeti*; (H) *Trichaitophorus koyaensis* SEM; (I) *Lambersaphis pruinosae*; (J) *Pseudopterocomma hughi* LM; (K) *Atheroides serrulatus*; (L) *Chaetosiphella stipae*; (M) *Laingia psammae*; (N) *Sipha* (*Rungsia*) *maydis* Chaitophorinae SEM.

5 Secondary rhinaria on antennal segment III: (0) absent; (1) present
6 Setae on antennal segment III: (0) present; (1) absent
7 Setae on antennal segment III: (0) equal or shorter than diameter of antennal segment III; (1) longer than diameter of antennal segment III
8 Diameter of primary rhinarium on penultimate segment: (0) equal or larger than width of antennal segment; (1) smaller than width of antennal segment
9 Longest basal seta: (0) equal or shorter than width at base; (1) longer than width at base
10 Processus terminalis: (0) short, shorter or a bit longer than the base; (1) long, much longer than the base
11 Compound eyes: (0) present; (1) absent
12 Compound eyes: (0) normal (Fig 3B); (1) placed on lateral, prominent extensions (Fig 3A)
13 Triommatidium: (0) well developed; (1) weakly developed
14 Segment II of rostrum: (0) without wishbone-shaped arch; (1) with wishbone-shaped arch
15 Apical segment of rostrum: (0) short, blunt; (1) long, stilleto-shaped
16 Connection of head with prothorax: (0) not fused; (1) fused
17 Dorsal setae on body: (0) with only pointed apices; (1) with variable shaped apices
18 Dorsal cuticle: (0) reticular or spinulose structures present; (1) smooth
19 Sclerotization of the abdominal tergum (0) membranous; (1) membranous with slcerites/scleroites; (2) slerotized
20 Dorsal abdominal tubercles: (0) absent; (1) present
21 Legs: (0) not reduced; (1) more or less reduced
22 Tibial setae: (0) equal or shorter than diameter of tibiae; (1) longer than diameter of tibiae
23 Spinules on distal part of tibiae: (0) absent (Fig 5G–5N); (1) present (Fig 5D–5F)

**Fig 6:**
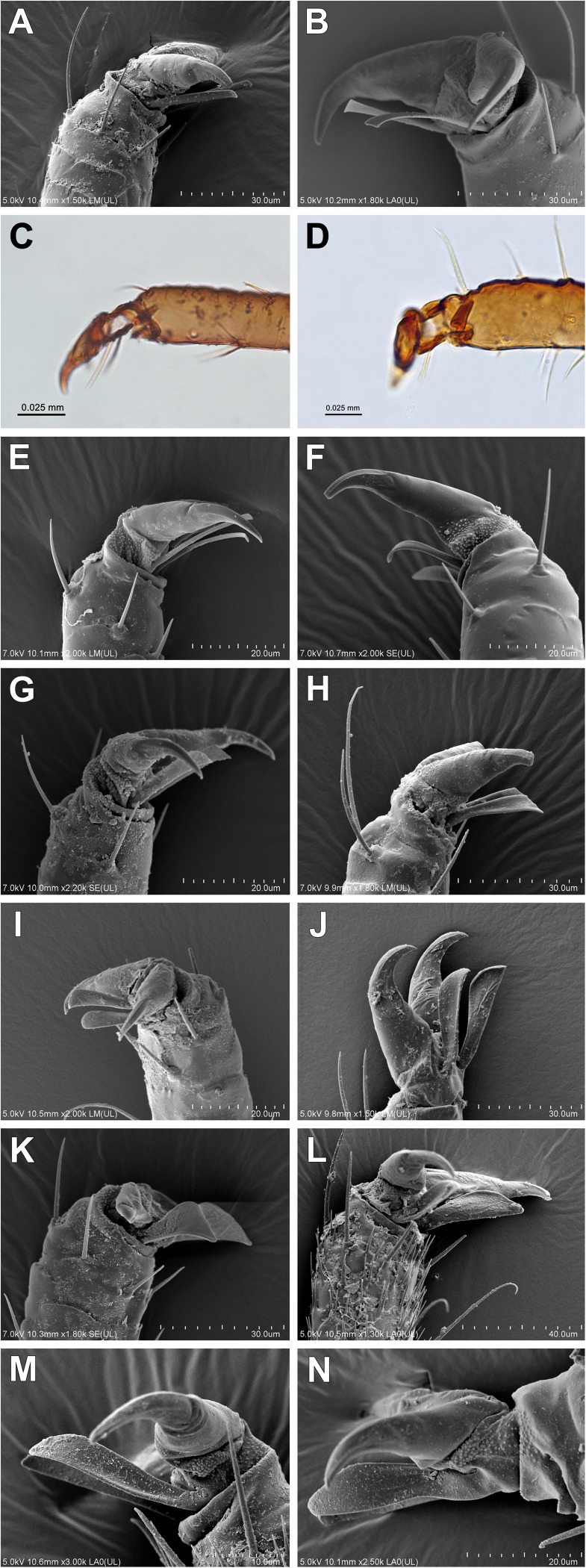
Shape of empodial setae. (A-F) Pointed (apterous viviparous females): (A) *Drepanosiphoniella aceris* Drepanosiphinae; (B) *Chaitophorus populeti* SEM; (C) *Lambersaphis pruinosae*; (D) *Pseudopterocomma hughi* LM; (E) *Laingia psammae*; (F) *Sipha* (*Rungsia*) *maydis* Chaitophorinae SEM. (G-I) Narrow spatulate (apterous viviparous females): (G) *Atheroides serrulatus*; (H) *Chaetosiphella stipae*; (I) *Trichaitophorus koyaensis* Chaitophorinae SEM. (J-N) Wide spatulate (J) *Periphyllus testudinaceus*; (K) *Caricosipha paniculatae* Chaitophorinae SEM; (L) *Drepanosiphum platanoidis*; (M) *Drepanaphis acerifoliae*; (N) *Yamatocallis tokyoensis* Drepanosiphinae SEM.

24 Ventral setae on the I tarsal segment: (0) 7–6; (1) 5–3
25 Dorsal setae on the I tarsal segment: (0) absent; (1) present
26 Empodial setae: (0) pointed (Fig 6A–6F); (1) narrow spatulate (Fig 6G–6I); (2) wide spatulate (Fig 6J and 6K)

**Fig 7:**
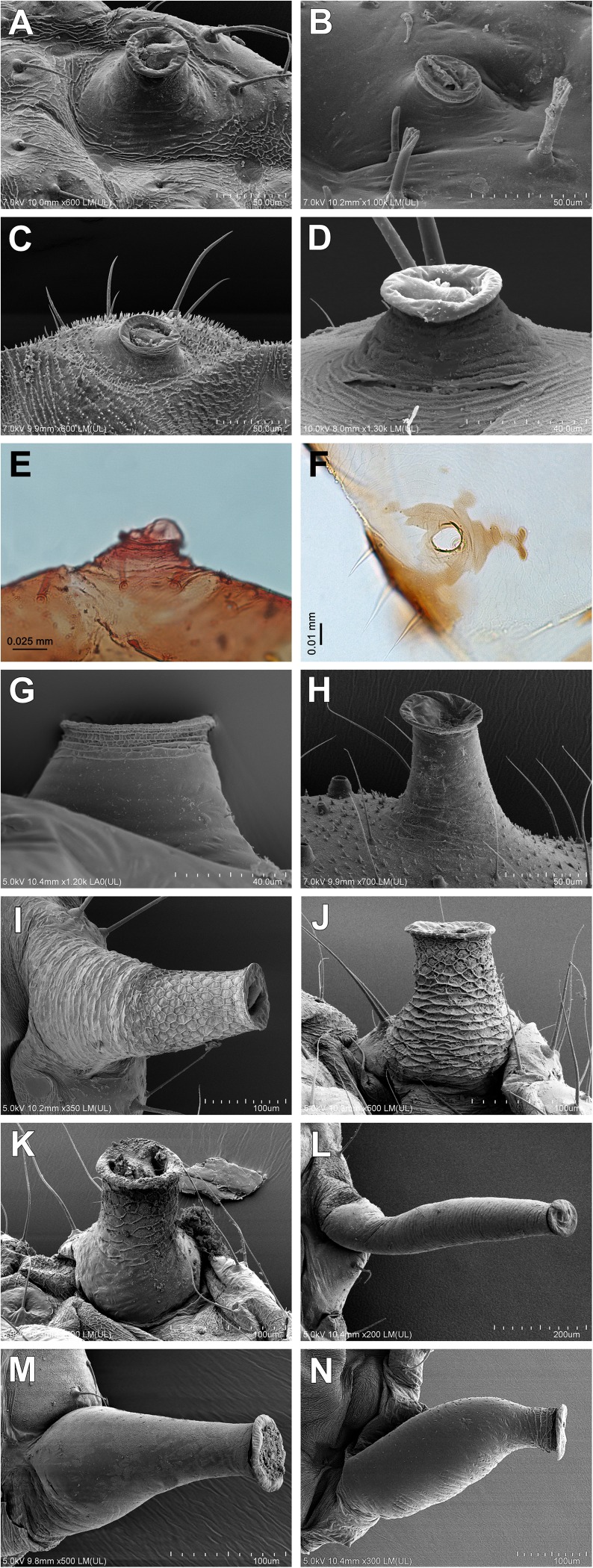
Shape of siphunculi. (A-F) Pore-shaped (apterous viviparous females): (A) *Atheroides serrulatus*; (B) *Chaetosiphella stipae*; (C) *Laingia psammae*; (D) *Sipha* (*Rungsia*) *maydis* SEM; (E) *Lambersaphis pruinosae*; (F) *Pseudopterocomma hughi* LM. (G, H) Low conical (apterous viviparous females): (G) *Trichaitophorus koyaensis*; (H) *Caricosipha paniculatae* SEM. (I-K) Elevated conical (apterous viviparous females): (I) *Periphyllus testudinaceus*; (J) *Chaitophorus populeti* Chaitophorinae; (K) *Drepanosiphoniella aceris* Drepanosiphinae SEM. (L-N) Elongated (alate viviparous females): (L) *Drepanosiphum platanoidis*; (M) *Drepanaphis acerifoliae*; (N) *Yamatocallis tokyoensis* Drepanosiphinae SEM.

27 Siphunculi: (0) present; (1) absent
28 Localization of siphunculi: (0) on abdominal segment V; (1) on abdominal segment VI
29 Siphunculi: (0) porous; (1) elevated
30 Shape of siphunculi: (0) pore-shaped (Fig 7A–7F); (1) low conical (Fig 7G and 7H); (2) elevated conical (Fig 7J and 7K); (3) elongated

31 Reticulation on siphunculi: (0) without reticulation; (1) with reticulation
32 Cauda: (0) visible; (1) covered by abdominal segment VIII
33 Shape of cauda: (0) knobbed; (1) broadly rounded; (2) tonque-shaped
34 Anal plate: (0) broadly rounded; (1) bilobed
35 Rudimentary gonapophyses: (0) 4; (1) 3; (2) 2
36 Wax glandular plates: (0) absent; (1) present

#### Alate viviparous females

**Fig 8:**
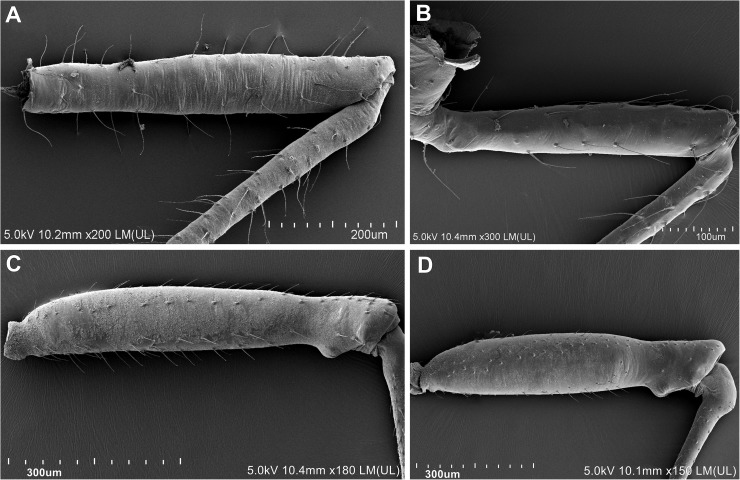
Character of fore femora. (A,B) Not enlarged (A) *Chaitophorus populeti* Chaitophorinae; (B) *Drepanosiphoniella aceris* Drepanosiphinae. (C, D) Enlarged (C) *Drepanaphis acerifoliae*; (D) *Yamatocallis tokyoensis* Drepanosiphinae SEM.

37 Type of body: (0) oval or pear-shaped; (1) slender
38 Frons: (0) straight; (1) with lateral or frontal tubercles
39 Number of antennal segments: (0) 6; (1) 5; (2) 5 or 4
40 Rhinaria: (0) ciliated (Fig 4A–4C); (1) not ciliated
41 Secondary rhinaria on antennal segment III: (0) absent; (1) present
42 Secondary rhinaria on antennal segment III: (0) ring like; (1) transverse oval (2) rounded
43 Secondary rhinaria on antennal segment III: (0) numerous, distributed over most of the length of the segment, in a few rows; (1) not numerous, confined to the basal 2/3 of the segment, in one row; (2) numerous, distributed over most of the length of the segment, in one row
44 Secondary rhinaria on antennal segment III: (0) distributed over the whole length of the segment; (1) distributed over up to half the length of the segment
45 Setae on antennal segment III: (0) present; (1) absent
46 Setae on antennal segment III: (0) equal or shorter than diameter of antennal segment III; (1) longer than diameter of antennal segment III
47 Diameter of primary rhinarium: (0) equal or greater than width of its antennal segment; (1) smaller than width of its antennal segment
48 Longest basal seta: (0) equal or shorter than width at base; (1) longer than width at base
49 Accessory rhinaria on BASE: (0) far from primary rhinarium; (1) close to primary rhinarium
50 Processus terminalis: (0) short, shorter or a bit longer than the base; (1) long, much longer than the base
51 Compound eyes: (0) normal; (1) placed on lateral, prominent extensions
52 Triommatidium: (0) well developed; (1) weakly developed
53 Segment II of rostrum: (0) without wishbone-shaped arch; (1) with wishbone-shaped arch
54 Apical segment of rostrum: (0) short, blunt; (1) long, stilleto-shaped
55 Dorsal setae on the body: (0) with only pointed apices; (1) with variable shaped apices
56 Dorsal cuticle: (0) reticular or spinulose structures present; (1) smooth
57 Sclerotization on the abdomen: (0) membranous; (1) membranous with slcerites/scleroites; (2) slerotized
58 Dorsum: (0) without tubercles; (1) with tubercles
59 Fore or mid femora: (0) normal (Fig 8A and 8B); (1) enlarged (Fig 8C and 8D)

60 Tibial setae: (0) equal or shorter than diameter of tibiae; (1) longer than diameter of tibiae
61 Rastral spines: (0) absent; (1) present (Fig 5A–5C)
62 Spinules on distal part of tibiae: (0) absent; (1) present (Fig 5A–5C)
63 Ventral setae on the I tarsal segment: (0) 7–6; (1) 5–3
64 Dorsal setae on the I tarsal segment: (0) absent; (1) present
65 Empodial setae: (0) pointed; (1) narrow spatulate; (2) wide spatulate (Fig 6L–6N)
66 Shape of fore wings: (0) normal, with the apex broadly rounded; (1) long and narrow
67 Number of branches of media: (0) 3; (1) 2; (2) 1
68 Origin of cubitus veins: (0) fused at base; (1) close to each other; (2) far from each other
69 Pigmentation on fore wings: (0) unpigmented; (1) pigmented
70 Fore wings when pigmented: (0) wholly pigmented; (1) wing veins or their apices conspicuously bordered with dark pigment
71 Siphunculi on abdominal segment: (0) V; (1) VI
72 Siphunculi: (0) porous; (1) elevated
73 Shape of siphunculi: (0) pore-shaped; (1) low conical; (2) elevated conical; (3) elongated (Fig 7L and 7M)
74 Reticulation on siphunculi: (0) absent; (1) present
75 Cauda: (0) visible; (1) covered by abdominal segment VIII
76 Shape of cauda: (0) knobbed; (1) broadly rounded; (2) tonque-shaped
77 Anal plate: (0) broadly rounded; (1) bilobed
78 Rudimentary gonapophyses: (0) 4; (1) 3; (2) 2
79 Wax glandular plates: (0) absent; (1) present

#### Oviparous females

80 Pseudosensoria of oviparae: (0) circular with small central pore; (1) circular; (2) 8-shaped; (3) irregular
81 Last abdominal segment of oviparae: (0) normal; (1) extended

#### Males

82 Male genitalia: (0) parameres not modified; (1) parameres modified

#### Biology

83 Life cycle: (0) monoecious; (2) heteroecious
84 Viviparous females: (0) all alate; (1) alate and apterous
85 Fundatrices: (0) morphologically similar to apterous viviparous females; (1) morphologically not similar to apterous viviparous females, thick and large with relatively short antennae and processus terminalis
86 Male: (0) alate; (1) apterous; (2) alate and apterous
87 Morphologically specialized aestivating nymphs: (0) absent; (1) present
88 Host plants: (0) coniferous; (1) deciduous trees; (2) herbaceous plants; (3) grasses or sedges
89 Attendance by ants: (0) no; (1) yes
90 Gall induction: (0) no; (1) yes

#### Phylogenetic analyses

**Molecular dataset**. Phylogenetic inferences were obtained individually for each of the genes and for joined alignments using Bayesian inference (BI) and Maximum likelihood (ML). BI was run using MrBayes 3.1 [69–70] with 1 cold and 3 heated Markov chains for 10 000 000 generations, and trees were sampled every 1000th generation. Each simulation was run twice. Convergence of Bayesian analyses was estimated using Tracer v. 1.5.0 [71], all trees sampled before the likelihood values stabilized were discarded as burn-in, and the remainder used to reconstruct a 50% majority rule consensus tree. ML analyses were implemented in RAxML 7.2.6 [72–73] with the GTR+I+G model and the same model parameters as in the Bayesian analyses. Branch support for ML analyses was assessed by bootstrapping with 1000 replicates. All trees were visualized using TreeView 1.6.6 [74].

#### Morphological dataset

Morphological analyses were rooted with *H*. *betulinus*. Datasets were analyzed with MP under equal weights using TNT v1.1 [75]. New technology searches were applied consisting of 10 000 random addition sequence replicates and TBR. (TBR) branch swapping. Clade support was assessed with 1000 replicates of the bootstrap [76]. Bayesian analyses were also performed in the same way as described above.

## Results

### Molecular data analyses

**Fig 9:**
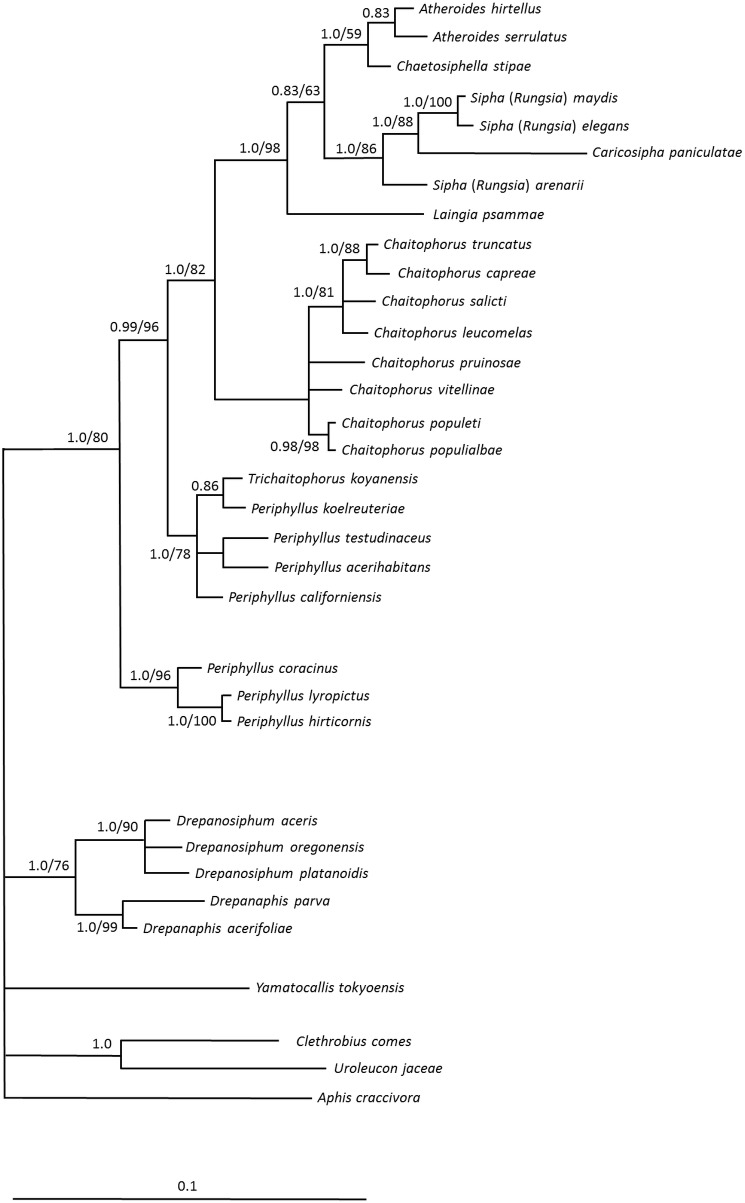
Phylogenetic tree of Chaitophorinae and Drepanosiphinae and outgroups. Phylogenetic tree of Chaitophorinae and Drepanosiphinae and outgroups based on the *EF-1α* gene and Bayesian inference. Numbers indicate posterior probabilities of Bayesian inference (shown only when above 0.80) and bootstrap values for nodes with the same topology on maximum likelihood tree (shown only when above 50%).

**Fig 10:**
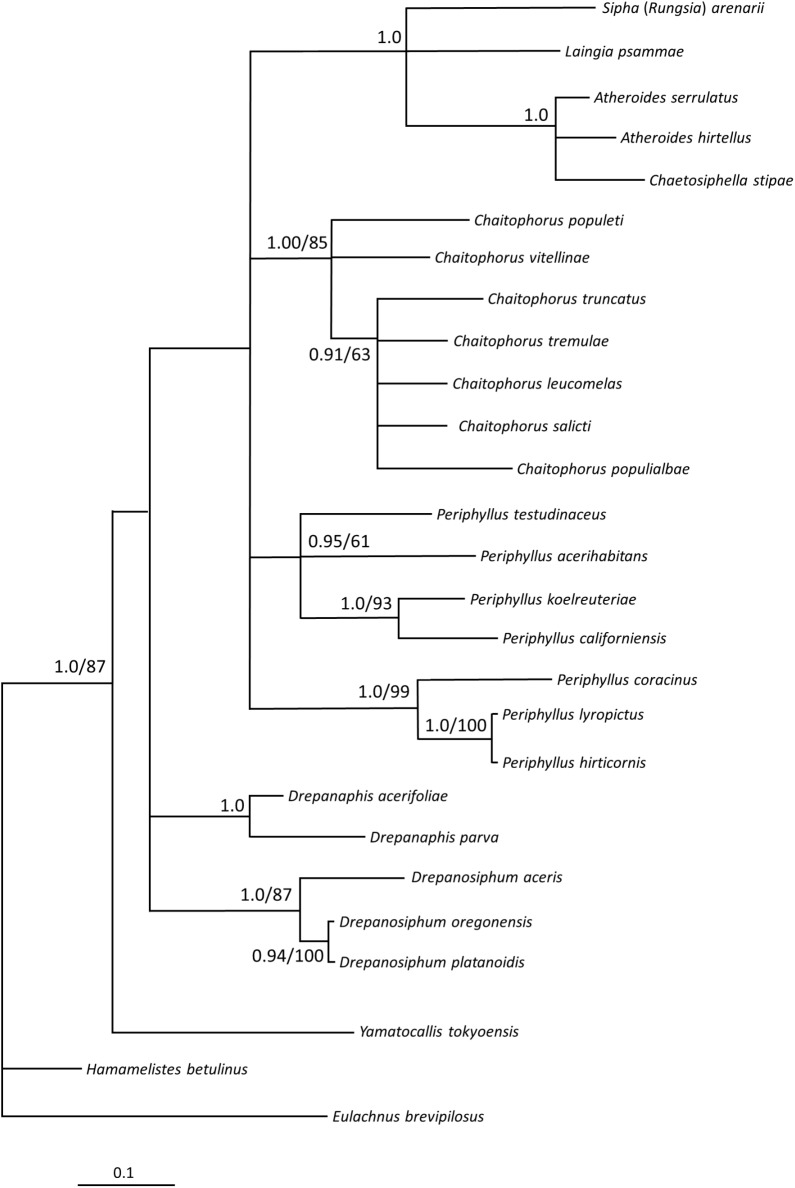
Phylogenetic tree of Chaitophorinae and Drepanosiphinae and outgroups. Phylogenetic tree of Chaitophorinae and Drepanosiphinae and outgroups based on the *COI* gene and Bayesian inference. Numbers indicate posterior probabilities of Bayesian inference (shown only when above 0.80) and bootstrap values for nodes with the same topology on maximum likelihood tree (shown only when above 50%).

**Fig 11:**
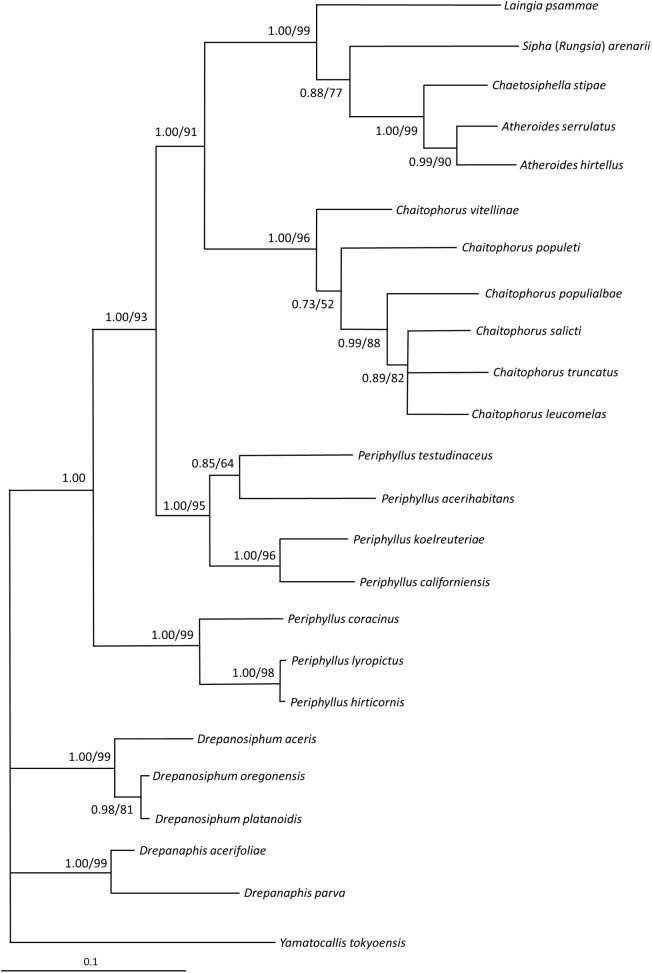
Phylogenetic tree of Chaitophorinae and Drepanosiphinae and outgroups. Phylogenetic tree of Chaitophorinae and Drepanosiphinae based on the joined *COI* and *EF-1α* genes and Bayesian inference. Numbers indicate posterior probabilities of Bayesian inference (shown only when above 0.80) and bootstrap values for nodes with the same topology on maximum likelihood tree (shown only when above 50%).

The topologies of both, Bayesian and Maximum Likelihood trees generated for *COI* gene, *EF-1α* gene and for joined sequences were congruent for particular markers, therefore only BI topologies are presented on figures (Figs 9–11), with added values of bootstraps from analogous nodes on ML trees.

Analysis of *EF-1α* gene resolved five clades with most nodes highly supported (Fig 9). The genus *Periphyllus* was recovered as paraphyletic. Two clades included the following species of *Periphyllus*: i) European species *P*. *coracinus* (Koch, 1854), *P*. *lyropictus* Kessler, 1886, *P*. *hirticornis* Walker, 1848 and ii) Asiatic species *P*. *koelreuteriae* (Takahashi, 1919), *P*. *californiensis* (Shinji, 1917), *P*. *acerihabitans* Zhang, 1982, *P*. *testudinaceus* (Fernie, 1852). The latter includes also *Trichaitophorus koyaensis* Takahashi, 1961. The species belonging to the genus *Chaitophorus* formed a monophyletic group sister to the species in the tribe Siphini. Among *Chaitophorus* species were two groups with strong support: i) *Ch*. *populialbae* (Boyer de Fonscolombe, 1841) and *Ch*. *populeti* (Panzer, 1804) and ii) *Ch*. *leucomelas* Koch, 1854, *Ch*. *salicti* (Schrank, 1801), *Ch*. *capreae* (Mosley, 1841) and *Ch*. *truncatus* Hausmann, 1802. Within the subfamily Drepanosiphinae were two groups: i) *Drepanosiphum aceris* Koch, 1855, *D*. *oregenensis* Granovsky, 1939, *D*. *platanoidis* and ii) *Drepanaphis parva* Smith 1941 and *D*. *acerifloliae* (Thomas, 1878). *Yamatocallis tokyoensis* (Takahashi, 1923) was found to be an independent phylogenetic lineage of the remaining Drepanosiphinae and the clade constituted by Chaitophorinae.

*COI* was sequenced for relatively fewer taxa than *EF-1α*. The *COI* tree (Fig 10) confirmed paraphyly of *Periphyllus* with the same species clustering as in the *EF-1α* tree (data for *T*. *koyaensis* not included). The monophyly of the genus *Chaitophorus* was also supported by the *COI* tree, however this genus was clustered with Asiatic *Periphyllus*, but with rather weak support. Siphini aphids, based on mtDNA, seemed to be sister to the Chaitophorini clade but with very weak support (0.37 PP). Subfamily Drepanosiphinae was again found to be sister to the Chaitophorini-Siphini clade and divided into two subclades, much as in *EF-1α* tree. *Yamatocallis tokyoensis* was most distant in *COI* tree and formed a third lineage next to the Drepanosiphinae, Siphini and Chaitophorini lineages.

Phylogenetic trees constructed on joined datasets (*COI* and *EF-1α* genes) (Fig 11) showed generally similar topologies like abovementioned trees. Monophyly of Drepanosiphinae could not be confirmed on phylogenetic trees based on joined sequences. On the other hand Chaitophorini-Siphini was very well supported (1.00 PP). Moreover, within this clade, three phylogenetic lineages were clearly confirmed (all with 1.00 PP): i) European species of *Periphyllus*, ii) Asiatic species of *Periphyllus* and iii) *Chaitophorus* with Siphini. The third clade constituted with also two well supported lineages (both 1.00 PP): members of *Chaitophorus* and sister to them–Siphini species.

### Morphological data analyses

**Fig 12:**
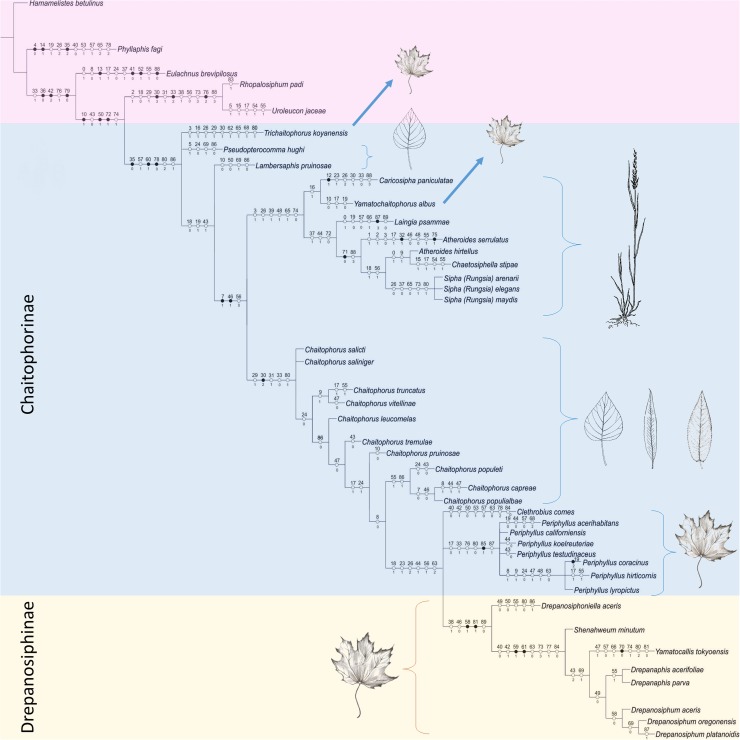
Phylogenetic tree of Chaitophorinae and Drepanosiphinae and outgroups. Strict consensus of the six most parsimonious trees resulting from the analysis of morphological dataset under equally weighted parsimony. Numbers above and below circles on the branches indicate character and state numbers, respectively. White and black circles represent homoplasious and nonhomoplasious states, respectively.

The morphological analysis includes representatives of all genera of Chaitophorinae (with exception of *Chaitogenophorus*) and Drepanosiphinae. The morphological analysis conducted in TNT (consensus on six trees Fig 12) corroborate monophyly of Siphini and paraphyly of *Periphyllus*, with the same subdivision of the studied taxa studied as in the earlier analysis. However, the species belonging to the genus *Chaitophorus*, on the basis of one synapomorhy (the shape of siphunculi), were clustered with the genus *Periphyllus*. Both were sister to the species from the tribe Siphini. *L*. *pruinosae* and *P*. *hughi* (not included in molecular analysis) formed independent lineages with *T*. *koyaensis* as a sister to the latter lineage, whereas *Y*. *albus* (all Chaitophorini) was nested inside Siphini. Drepanosiphinae, on the basis on two synapomorphies, formed a clade independent of the remaining taxa with *Y*. *tokyoensis* nested inside this unit. Bayesian tree resulting from the Bayesian analysis of morphological dataset with weak of supports makes its interpretation too speculative (S1 Fig).

## Discussion

Currently aphids are divided into 24 subfamilies [2]. However, comparisons of the endosymbiotic bacterial phylogeny [77–78,36,] with the morphology of viviparous females [79–81,50] and molecular-based aphid phylogenies [33–35] indicate phylogenetic incongruence at higher taxonomic levels with no full set of mutual relationships within the group. Such incongruence also occurs at lower taxonomic levels. Most of the attempts to identify phylogenetic relationships based on molecular data are for Aphidinae [53–55,58,82–83], Eriosomatinae [59,84–87], Hormaphidinae [88–91] and Lachninae [57,92–95]. Analyses based on a total-evidence dataset are rather rare [37,91].

### Comparison of the tribal and generic relationships with historical classification of Chaitophorinae

The major point of discordance between our molecular data and the classical taxonomy of Chaitophorinae is the tribal division of this subfamily. Analysis of the *EF-1α* gene and joined datasets of *COI* and *EF-1α* genes, highly supported closer relationships between *Chaitophorus* and the genera included in Siphini. The *COI* trees are much less resolved as it constitute of several parallel phylogenetic lineages which group independently: Siphini, *Chaitophorus*, European *Periphyllus* and Asiatic *Periphyllus* (Fig 10). However, in all molecular-based trees Chaitophorini do not form a monophyletic clade.

Another result that contrasts with historic views is the position of the genus *Periphyllus*. Taxonomists have found this highly polymorphic genus consisting of 42–44 species [4] confusing for many years. In the life cycle of most species of this genus about 15 types of morphs (including aestivating highly specialized first instar nymphs) among the normal generations of individuals have been identified [25]. Next to these unique biological features, recognition characters for this genus includes abdominal tergum membranous in wingless viviparous females, dorsal cuticle smooth, presence of spinules on distal part of tibiae and conical siphunculi with distinct reticulation. Our analyses of both molecular and morphological data (Figs 9–12) show that this taxon is paraphyletic. The European species analyzed (*P*. *coracinus*, *P*. *lyropictus*, *P*. *hirticornis*) were clustered together as were the Asiatic species (*P*. *koelreuteriae*, *P*. *californiensis*, *P*. *acerihabitans*, *P*. *testudinaceus*). In the latter clade (and within the genus as a whole) *P*. *californiensis* and *P*. *testudinaceus* are the most widely distributed in the Northern Hemisphere and recorded from the most species of maples [5]. Interestingly, both species are abundant on the Pacific Coast and rarely collected along the Atlantic Coast and elsewhere in eastern North America [25]. The diameter of primary rhinarium on penultimate antennal segment, length of basal seta of antennal segment VI and number of ventral setae are, among others, morphological characters clearly distinguishing two clades of *Periphyllus*. Our study points to a potentially interesting feature of *Periphyllus* but firm conclusions about the evolutionary patterns of species in this genus cannot yet be made because our analysis did not include members of the genus native to Central Asia nor the Nearctic.

Among all the taxa of Chaitophorinae studied *Trichaitophorus koyaensis* seems to be the most closely related to *Periphyllus* (Fig 9). *Trichaitophorus* seems to be even more polymorphic than *Periphyllus*, with complicated life cycles and numerous intermediate morphs characterized by variation in setal length and shape, as well as winged females strongly resembling *Periphyllus* [23, 96–97]. On the other hand wingless viviparous females are characterized by unique characters like 5- or 4-segmented antennae, fused head and pronotum, very short and sparse dorsal setae in exception of lanceolate marginal ones, as well as slightly elevated siphunculi. Further field studies, in conjunction with laboratory analysis of additional species, morphs and genes, may ultimately show that the two genera are not justified as currently structured.

*Chaitophorus* is the largest genus within the Chaitophorinae, recognized by dorsum sclerotized with a distinct reticulation and, with some exceptions (e.g. *Ch*. *populicola* Thomas, 1878), a knobbed cauda. Our analysis supports the monophyly of this genus, however the hypothesis that willow-feeding species are monophyletic within *Chaitophorus* and separate from poplar-feeding species is not supported. Moreover, the species with a certain feeding position i.e. leaf-feeding species versus petiole-feeding species do not constitute clear clades as well as ant-attended versus not ant-attended species. Shingleton and Stern [98] constructed a molecular phylogeny of 15 species of *Chaitophorus* based on mtDNA sequences and obtained similar results. Our research based on a different set of species and molecular markers is congruent with the more general theory that during evolution *Chaitophorus* has several times switched host plants (from poplars to willows), feeding position and ant tending [98]. The high plasticity of this genus is also reflected in variation in the shape and thickness of the dorsal setae, which is correlated with the seasonal development and distribution of particular species [26–27,99]. *Chaitophorus*, with its ability to switch host plants and feeding position in the course of evolution, is considered to be the ancestral form for the Chaitophorinae [8,29–30]. However, lack of fossil evidence makes discussion of the origin of the subfamily somewhat hypothetical.

As our research are based on a different set of species in molecular (eight of twelve total genera) and morphological (eleven of twelve total genera) analysis is difficult to directly compare the obtained results. In particular, the position of *Lambersaphis* and *Pseudopterocomma*, traditionally included in the Chaitophorini, is not well-justified. The forewing veins of the species in both genera characteristically have a pigmented border like the Nearctic species of *Chaitophorus* and similar affinities to host plants, however they are placed in the most isolated positions in the cladogram (Fig 12). Moreover, *Pseudopterocomma* is characterized by unique set of features like processus terminalis covered in numerous, fine, hair-like setae, presence of secondary rhinaria on antennal segment III and IV both in winged and wingless viviparous females, as well as porous, not reticulated siphunculi. Similarly, *Lambersaphis* is characterized by very short processus terminalis, slightly elevated siphunculi without reticulation and short, needle-like dorsal setae. The position of *Yamatochaitophorus albus* (Takahashi, 1961) also remains unclear, as this species was nested inside Siphini clade (Fig 12). Traditionally this genus is placed close to *Trichaitophorus*, as these two genera share similar morphological characters and differ by pattern of dorsal chaetotaxy.

### Relationships within Drepanosiphinae

Our analyses of mitochondrial and nuclear genes resulted in a stable phylogenetic reconstruction with well-supported clades (Figs 9–11). In the genus *Drepanosiphum*, *D*. *oregonensis* and *D*. *platanoidis* were clustered together and sister to *D*. *aceris*. *Drepanaphis* species (*D*. *parva* and *D*. *acerifoliae*) also were clustered together and sister to *Drepanosiphum*. Although morphological analysis comprises representatives of all genera of Drepanosiphinae, the combination of synapomorphies: enlarged fore (Fig 8C and 8D) or mid femora and presence of rastral spines on hind tibiae (Fig 5A–5C), also supports this division (Fig 12). The only exception is the position of *Yamatocallis tokyoensis*. In the molecular analysis this species was placed in an independent lineage far from the remaining Drepanosiphinae. In our morphological analysis, on the other hand, *Y*. *tokyoensis* is nested within Drepanosiphinae, with a sister relation to species of *Drepanaphis* (Fig 12), which is congruent with traditional taxonomy. Its position is supported by one synapomorhy: pigmented forewings (Fig 2D). Originally, members of *Yamatocallis* were placed in the Nearctic *Drepanaphis* [100–101], as in general appearance species of these genera are similar. However, a combination of characters like accessory rhinaria located close to the major rhinarium, abdomen without dorsal tubercles and elongated siphunculi with reticulated apex (Fig 7N), clearly distinguish *Yamatocallis* from this genus and other taxa of Drepanosiphinae. In addition, Fukatsu [102] reports the secondary intracellular symbiotic bacterium YSMS in *Y*. *tokyoensis* (and *Y*. *hirayamae* Matsumura, 1917), which is treated as conserved throughout the evolution of the genus. Our molecular analyses show that *Yamatocallis* is farther from other species of this subfamily than previously thought [4,22]. The presence of this unique secondary mycetocyte symbiont, whose time of acquisition was estimated as the Miocene, may also indicate the separation of this genus. In this epoch dramatic geological and climatic changes took place. Isolation of Eastern Asia by the uplift of the Himalayas fits with the hypothesis that *Yamatocallis* was isolated from the other Palaearctic and Nearctic species of Drepanosiphinae.

### Drepanosiphinae versus Chaitophorinae

Despite the increased use of molecular methods in phylogenetic analyses, morphology continues to play a significant role in the understanding of the evolutionary biology and systematics of many groups of organisms [103]. According to Quednau’s hypothesis [50], based on morphological and biological characters, Drepanosiphinae evolved as a sister group of the Chaitophorinae and probably have a common ancestral form in *Taiwanaphis*- or *Monaphis*-like aphids. Close relationships between these subfamilies are reflected in the similarities in their morphology (i.e. absence of sclerotisation of segment II of the rostrum, absence of wax glands), anatomy (i.e. gastrointestinal tract without a filter chamber [104]), similar internal male reproductive system [31] and male genitalia [105] or bionomy (associations with host plants, similar type of summer diapause). According to this hypothesis (also indicated in Fig 12), during their evolutionary scenario, representatives of Drepanosiphinae probably lost some apomorphic features and became *Periphyllus*-like (Chaitophorinae). The intermediate characters between species of Chaitophorinae and Drepanosiphinae occur in the representatives of the genus *Drepanosiphoniella*, i.e., presence of apterous morphs in the life cycle (Fig 2C), nude primary sensoria, (Fig 4D) or lack of leaping legs (Fig 8B), features common in most Chaitophorinae. As representatives of *Drepanosiphoniella* were not included in the molecular studies, the position of this genus can only be discussed based on the morphological and biological characters. Currently fossils of about eight genera and 20 species of Drepanosiphinae are described (Eocene, Middle Miocene), but only one fossil of Chaitophorinae (*Chaitophorus salijaponicus niger* Mordvilko, 1929) is known from the Late Pliocene-Early Pleistocene (Peary Land, Greenland) [106]. Its also supports the hypothesis that Drepanosiphinae are an independent lineage within drepanosiphine aphids (sensu Quednau, [50]), which is also congruent with the biological data. At least *Drepanosiphum* has several highly specialized parasitoids whose life cycles are closely synchronized with the life cycle of the aphid-host [107] and this relationship developed a long time ago in parallel during the evolution of both insects [48].

## Conclusions

The generally accepted view of the classification of Chaitophorinae features the strict subdivision of two bionomic groups–monocotyledonous feeding Siphini and deciduous tree or shrub feeding Chaitophorini. Commonly accepted diagnosis define the Chaitophorini includes 6-segmented antennae and elevated siphunculi with reticulated apices. Due to this fact, *Lambersaphis* and *Pseudopterocomma* should be excluded from the Chaitophorini, as both have rather short, even pore-shaped siphunculi without reticulation and in *Pseudopterocomma* the antennae of the wingless viviparous females are 6- or 5-segmented, characters more closely fitting Siphini. Genera *Trichaitophorus* and *Yamatochaitophorus*, both included in Chaitophorini, are characterised by 6- or 5 (4)-segmented antennae and short siphunculi in wingless viviparous females whereas winged females have 6-segmented antennae and elevated and clearly reticulated siphunculi thereby strongly resembling *Periphyllus*. Therefore, the number of antennal segments and reticulation of siphunculi should not to be treated as good characters for tribal subdivision.

Our molecular analyses, supported by morphological and biological data, revealed at least four clades within Chaitophorinae: (1) Siphini closely related to (2) *Chaitophorus*, (3) paraphyletic *Periphyllus* with *Trichaitophorus* (and *Yamatochaitophorus*) and (4) the most distant *Lambersaphis* and *Pseudopterocomma*. All of these genera share the presence of four gonaphophyses, which is also the synapomorphy for Chaitophorinae as well as the entire anal plate.

The relationships within Drepanosiphinae are much clearer, with the exception of *Yamatocallis*, which seems to be an independent lineage.

## Supporting information

S1 Table Voucher information and GenBank accession numbers for the sequenced data. (DOCX)

S2 Table Morphological data matrix part I. (DOCX)

S3 Table Morphological data matrix part II. (DOCX)

S1 Fig Bayesian tree resulting from the Bayesian analysis of morphological dataset. Numbers above each node indicate posterior probabilities (PP) values (shown only when above 0.80).
(TIF)
