# Supplementary material for: The relationships within the Chaitophorinae and Drepanosiphinae (Hemiptera, Aphididae) inferred from molecular-based phylogeny and comprehensive morphological data
Source: PLoS One. 2017 Mar 13;12(3):e0173608. doi: 10.1371/journal.pone.0173608 (PMC5348029; doi:10.1371/journal.pone.0173608)
Supplement: S1 Table — (DOCX) [file pone.0173608.s001.docx]

| **Subfamily** | **Species** | **Host plant** | **Location** | **Collection date and deposition** | **No. voucher** | **GB numbers**  **COI** | **GB numbers**  **EF** |
| --- | --- | --- | --- | --- | --- | --- | --- |
| Chaitophorinae | *Atheroides hirtellus* Haliday, 1839 | *Deschampsia caespitosa* | Poland  Piekary Śląskie | Jun.23,2009  UŚ | 61013 | KU570441 | HM988755^+^ |
| Chaitophorinae | *Atheroides serrulatus* Haliday, 1839 | *Festuca ovina* | Poland Dąbrowa Górnicza | Sep.29,2008  UŚ | 90863 | KU570450 | HM132094^+^ |
| Chaitophorinae | *Caricosipha paniculatae* Bӧrner, 1939 | *Carex brizoides* | Poland Głogów Małopolski | Jul.5,1998  UŚ | 98750 | - | HM132092^+^ |
| Chaitophorinae | *Chaetosiphella stipae* Hille Ris Lambers, 1947 | *Stipa capillata* | Poland Owczary | Sept.20,  2009; UŚ | 90962 | KU570440 | HM132095^+^ |
| Chaitophorinae | *Laingia psammae* Theobald, 1922 | *Calamagrostis epigejos* | Poland Katowice | Oct.10,2009  UŚ | 100979 | KU570438 | HM132093^+^ |
| Chaitophorinae | *Sipha* (*Rungsia*) *arenarii* Mordvilko, 1921 | *Leymus arenarius* | Poland Bukowno | May.17,  2009; UŚ | 50944 | KU570439 | HM132091^+^ |
| Chaitophorinae | *Sipha* (*Rungsia*) *elegans* Del Guercio, 1905 | *Arrhenatherum elatius* | Poland Dąbrowa Górnicza | Jun.13,2009  UŚ | 90613 | - | HM132089^+^ |
| Chaitophorinae | *Sipha* (*Rungsia*) *maydis* Passerini, 1860 | *Arrhenatherum elatius* | Poland Katowice | Jun.7,2007  UŚ | 70670 | - | HM132090^+^ |
| Chaitophorinae | *Chaitophorus capreae* (Mosley, 1841) | *Salix cinerea* | Poland Katowice | Aug.11,  2012; UŚ | 12811 | - | HM987561^+^ |
| Chaitophorinae | *Chaitophorus leucomelas* Koch, 1854 | *Populus tremula* | Poland Katowice | Sept.5,2009  UŚ | 90924 | KU570429 | KU571716 |
| Chaitophorinae | *Chaitophorus populialbae* (Boyer de Fonscolombe, 1841) | *Populus nigra* | Poland Katowice | Jul.26,2012  UŚ | 71276 | KU570434 | DQ493831* |
| Chaitophorinae | *Chaitophorus populeti* (Panzer, 1804) | *Populus nigra* | Poland Katowice | Jul.26,2012  UŚ | 71279 | KU570430 | KU571717 |
| Chaitophorinae | *Chaitophorus pruinosae* Narzikulov, 1954 | *Populus pruinosa* | Tadzikhistan Tigrova Balka | Jun.21,2014  UŚ | 61436 | - | KU571711 |
| Chaitophorinae | *Chaitophorus salicti* (Schrank, 1801) | *Salix cinerea* | Poland Katowice | Jun.9,2012  UŚ | 61226 | KU570431 | KU571710 |
| Chaitophorinae | *Chaitophorus saliniger* Shinji, 1924 | *Salix* sp. | China Beijing | Oct.9,2009  UŚ | 90109 | - | - |
| Chaitophorinae | *Chaitophorus tremulae* Koch, 1854 | *Populus tremula* | Poland Katowice | Aug.16,  2012; UŚ | 81231 | KU570433 | - |
| Chaitophorinae | *Chaitophorus truncatus* Hausmann, 1802 | *Salix* sp. | Poland Katowice | Sept.2,2011  UŚ | 91147 | KU570428 | KU571709 |
| Chaitophorinae | *Chaitophorus vitellinae* (Schrank, 1801) | *Salix* sp. | Poland Katowice | Aug.7,2011  UŚ | 81159 | KU570432 | KU571713 |
| Chaitophorinae | *Lambersaphis pruinosae* (Narzikulov, 1954) | *Populus pruinosa* | Tadzikhistan Dshilicul | Oct.25.1959  BMNH | BM1984-40 | - | - |
| Chaitophorinae | *Periphyllus acerihabitans* Zhang, 1982 | *Acer buergerianum* | Japan Tachibana | May.3,2011  UŚ | 51122 | KU570444 | KU571723 |
| Chaitophorinae | *Periphyllus californiensis* (Shinji, 1917) | *Acer palmatum* | Poland Kraków | May.11,  2009; UŚ | 50910 | KU570445 | KU571719 |
| Chaitophorinae | *Periphyllus coracinus* (Koch, 1854) | *Acer platanoides* | Poland Katowice | Apr.30,2009  UŚ | 40911 | KU570427 | KU571708 |
| Chaitophorinae | *Periphyllus hirticornis* Walker, 1848 | *Acer campestre* | Poland Gliwice | May.16,  2009; UŚ | 50916 | KU570426 | KU571724 |
| Chaitophorinae | *Periphyllus koelreuteriae* (Takahashi, 1919) | *Koelreuteria paniculata* | CHINA Tianjin | Nov.3, 2010  UŚ | 110267 | KU570443 | DQ493830* |
| Chaitophorinae | *Periphyllus lyropictus* Kessler, 1886 | *Acer platanoides* | Poland Bytom | Jun.27,2010  UŚ | 61027 | KU570425 | KU571707 |
| Chaitophorinae | *Periphyllus testudinaceus* (Fernie, 1852) | *Acer platanoides* | Poland Piekary Śląskie | May.4,2013 | 61238 | KU570442 | HM988757^+^ |
| Chaitophorinae | *Pseudopterocomma hughi* (MacGillivray, 1963) | *Populus tremuloides* | Canada Ontario | Aug.15,  1962; BMNH | BM1984-340 | - | - |
| Chaitophorinae | *Trichaitophorus koyaensis* Takahashi, 1961 | *Acer rufinerve* | Japan  Mt. Rocco | Aug.7,2012  UŚ | 81256 | - | KU571712 |
| Dreapnosiphinae | *Dreapanaphis acerifoliae* (Thomas, 1878) | *Acer* sp. | Spain Leon | May29,2011  UŚ | 51145 | KU570447 | KU571720 |
| Dreapnosiphinae | *Dreapanaphis parva* Smith 1941 | *Acer rubrum* | USA Washington DC | Jul.23,2014  UŚ | 71427 | KU570449 | KU571722 |
| Dreapnosiphinae | *Drepanosiphum aceris* Koch, 1855 | *Acer campestre* | Poland Pszczyna | Nov.9,2011  UŚ | 55921 | KU570435 | KU571721 |
| Dreapnosiphinae | *Drepanosiphum oregonensis* Granovsky, 1939 | *Acer pseudoplatanus* | Poland Katowice | Jul.16,2013  UŚ | 613122 | KU570436 | KU571715 |
| Dreapnosiphinae | *Drepanosiphum platanoidis* (Schrank, 1801) | *Acer platanoides* | Poland Katowice | Sept.10,  2011; UŚ | 91127 | KU570448 | KU571714 |
| Dreapnosiphinae | *Dreapnosiphoniella aceris* Davatchi, HRL& Remaudière, 1957 | *Acer cinerascens* | Iran Firouzabad | May.1,1959  MNHN | 55921 | - | - |
| Dreapnosiphinae | *Shenahweum minutum* (Davis, 1910) | *Acer saccharum* | USA  Illinois | Sept.9,1909  BMNH | BM1930-204 | - | - |
| Dreapnosiphinae | *Yamatocallis tokyoensis* (Takahashi, 1923) | *Acer mono* | Japan Tarumi | May.18,  2011; UŚ | 51167 | KU570446 | KU571718 |
| Aphidinae | *Aphis (Aphis) craccivora* Koch, 1854 | *Vicia cracca* | Poland Tarnowskie Góry | May.7,2000  UŚ | 20570 | - | EU358905* |
| Aphidinae | *Rhopalosiphum padi* Linnaeus, 1758 | *Padus avium* | Poland Dolistowo | Jun.15,1978  UŚ | 78615 | - | - |
| Aphidinae | *Uroleucon* (*Uromelan*) *jaceae* (Linnaeus, 1758) | *Centaurea* sp. | Slovakia Bratislava | Jul.6,1987  UŚ | 87066 | - | AF068470* |
| Callaphidinae | *Clethrobius comes* (Walker, 1848) | *Betula pendula* | Poland Katowice | Jun.2,2009  UŚ | 90660 | - | HM132097^+^ |
| Hormaphidinae | *Hamamelistes betulinus* Horvath, 1896 | *Betula pendula* | Poland Oborniki Śląskie | May.12,  2013; UŚ | 51328 | KU570437 | - |
| Lachninae | *Eulachnus brevipilosus* Bӧrner, 1940 | *Pinus nigra* | Poland Katowice | Oct.4,2014  UŚ | 10147 | KP637092^^^ | - |
| Phyllaphidinae | *Phyllaphis fagi* (Linnaeus, 1761) | *Fagus sylvatica* | Poland Katowice | May.12,  2013; UŚ | 51328 | - | - |

S1 Table. Collection data and Gen Bank accession numbers for the species studied

*GenBank accession; Reference sequences from previous studies: ^+^[37]; ^^^[96]
