# Supplementary figures and images for: The relationships within the Chaitophorinae and Drepanosiphinae (Hemiptera, Aphididae) inferred from molecular-based phylogeny and comprehensive morphological data
Source: PLoS One. 2017 Mar 13;12(3):e0173608. doi: 10.1371/journal.pone.0173608 (PMC5348029; doi:10.1371/journal.pone.0173608)

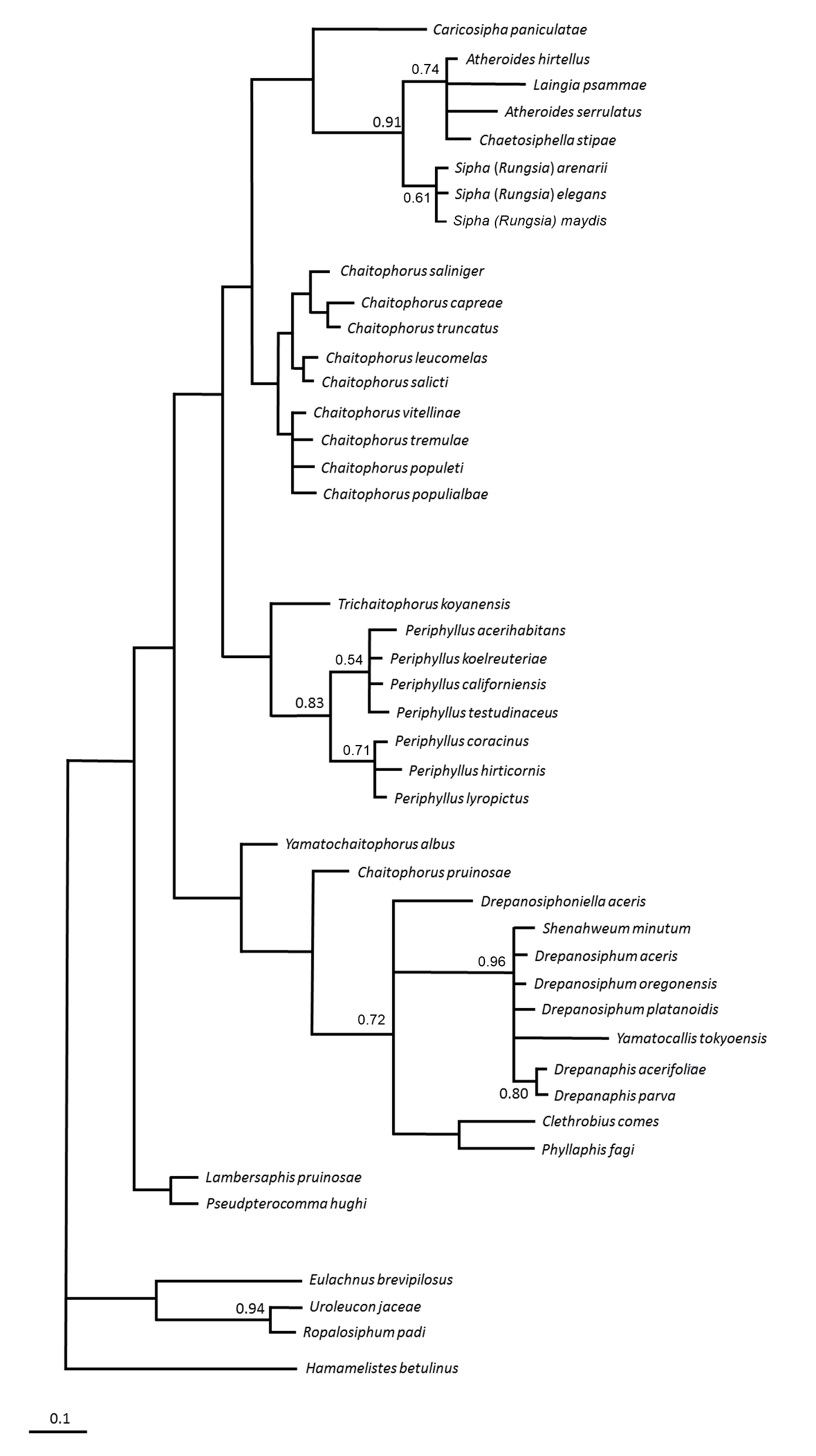

Supplement: S1 Fig — Numbers above each node indicate posterior probabilities (PP) values (shown only when above 0.80). (TIF) [file pone.0173608.s004.tif]
